# Does prazosin improve sleep disturbances and other trauma-related symptoms?

**DOI:** 10.1192/j.eurpsy.2025.1952

**Published:** 2025-08-26

**Authors:** T. M. Gómez Lezcano, C. Fernandez Natal, P. Campos Abraham, M. M. Gonzalvo Navarro, M. B. Sanjose, M. V. Sánchez, J. Correas Lauffer

**Affiliations:** 1 Psychiatry Department, Hospital Del Henares, Coslada, Madrid, Spain

## Abstract

**Introduction:**

Post-traumatic stress disorder (PTSD) is characterized by intrusive thoughts and flashbacks involving the traumatic event, hypervigilance and avoidance behavior. On the other hand, complex post-traumatic stress disorder (complex PTSD) can result from experiencing chronic trauma. It involves similar stress reponses, such as flashbacks, nightmares, and also avoidance of places and situations related to the traumatic event. Sleep disturbance is a central element of both disorders (PTSD and complex PTSD). While nightmares qualify as a re-experiencing symptom, initiation and maintenance of sleep stem from a hyperarousal state (Paiva et al., 2021). Evidence implies that treatment for PTSD-related sleep disturbance also improves other trauma-related symptoms. As pharmacological therapy, some clinicians use prazosin, an adrenergic inhibitor, which is a lipophilic drug originally developed to treat hypertension. It blocks α1 receptor sites and ameliorates the increase in noradrenergic activity in PTSD-diagnosed individuals (Lipinska G et al. 2016). Studies differ regarding the effectiveness of this drug to alleviate sleep disorders and other trauma-related symptoms (Bajor et al., 2022; Yücel et al., 2020; Zhang et al., 2020; Petrakis et al. 2016).

**Objectives:**

Through this case series study we want to understand the utility and effectiveness of prazosin in patients diagnosed with PTSD and complex PTSD for the improvement of nightmares and other PTSD-related symptoms.

**Methods:**

For this purpose we have reviewed in 10 patients with a diagnosis of either PTSD (4 out of 11) or complex PTSD (7 out of 11) the improvement of nightmares and other trauma-related symptoms, especially flashbacks. The patients indicated whether there was no, partial or total improvement of these symptoms. The doses used were between 1 to 2mg.

**Results:**

In this Case series study (n= 11), we focus on the reports from PTSD - and complex PTSD-diagnosed (4 out of 11 vs 7 out of 11) patients treated with prazosin regarding their sleep disturbance and other trauma-related symptoms.

Forty percent of the patients report a reduction in the frequency and intensity of nightmares, while 3 patients of the sample reported absolute extinction of nightmares.

Our findings reveal almost half of the patiens (4 out of 11) expressed as well a reduction in other PTSD symptoms, specifically in flashbacks.

We have not found a significant better outcome in either PTSD or complex PTSD- diagnosed patients. The entire sample were female patients.

Improvements in hypervigilance and avoidance behavior have hardly been reported in enrolled patients (3 out of 11).

**Conclusions:**

In our sample, the use of prazosin appears to be an acceptably good option, especially for the improvement of nightmares. Nevertheless, further studies should be conduced.

**Disclosure of Interest:**

None Declared

